# Evidence of traumatic brain injury in headbutting bovids

**DOI:** 10.1007/s00401-022-02427-2

**Published:** 2022-05-17

**Authors:** Nicole L. Ackermans, Merina Varghese, Terrie M. Williams, Nicholas Grimaldi, Enna Selmanovic, Akbar Alipour, Priti Balchandani, Joy S. Reidenberg, Patrick R. Hof

**Affiliations:** 1 Nash Family Department of Neuroscience, Icahn School of Medicine at Mount Sinai, One Gustave L Levy Place, New York, NY 10029 USA; 2 Friedman Brain Institute, Icahn School of Medicine at Mount Sinai, New York, NY USA; 3 Ronald M. Loeb Center for Alzheimer’s Disease, Icahn School of Medicine at Mount Sinai, New York, NY USA; 4 BioMedical Engineering and Imaging Institute, Icahn School of Medicine at Mount Sinai, New York, NY USA; 5 Center for Anatomy and Functional Morphology, Icahn School of Medicine at Mount Sinai, 1 Gustave L. Levy Place, Mail Box 1007, New York, NY 10029-6574 USA; 6 Department of Ecology and Evolutionary Biology, University of California Santa Cruz, Santa Cruz, CA 95060 USA; 7 University of Zurich, Rämistrasse 71, 8006 Zurich, Switzerland

**Keywords:** TBI, Chronic traumatic encephalopathy, CTE, Concussion, MRI, Tau protein

## Abstract

Traumatic brain injury (TBI) is a leading cause of neurologic impairment and death that remains poorly understood. Rodent models have yet to produce clinical therapies, and the exploration of larger and more diverse models remains relatively scarce. We investigated the potential for brain injury after headbutting in two combative bovid species by assessing neuromorphology and neuropathology through immunohistochemistry and stereological quantification. Postmortem brains of muskoxen (*Ovibos moschatus*, *n* = 3) and bighorn sheep (*Ovis canadensis*, *n* = 4) were analyzed by high-resolution MRI and processed histologically for evidence of TBI. Exploratory histological protocols investigated potential abnormalities in neurons, microglia, and astrocytes in the prefrontal and parietal cortex. Phosphorylated tau protein, a TBI biomarker found in the cerebrospinal fluid and in neurodegenerative lesions, was used to detect possible cellular consequences of chronic or acute TBI. MRI revealed no abnormal neuropathological changes; however, high amounts of tau-immunoreactive neuritic thread clusters, neurites, and neurons were concentrated in the superficial layers of the neocortex, preferentially at the bottom of the sulci in the muskoxen and occasionally around blood vessels. Tau-immunoreactive lesions were rare in the bighorn sheep. Additionally, microglia and astrocytes showed no grouping around tau-immunoreactive cells in either species. Our preliminary findings indicate that muskoxen and possibly other headbutting bovids suffer from chronic or acute brain trauma and that the males’ thicker skulls may protect them to a certain extent.

**Supplementary Information:**

The online version contains supplementary material available at 10.1007/s00401-022-02427-2.

## Introduction

Traumatic brain injury (TBI) is one of the main causes of neurological deficits and death worldwide, accounting for 2.5 million hospital admissions per year [1]. Human cranial anatomy is vulnerable to coup-contrecoup injuries and TBI is most often the result of a fall, motor vehicle collision, or firearm accident in the U.S. [1]. Military personnel and athletes are especially at risk and have become part of the mounting concern around TBI and its long-term effects [2]. Repetitive brain trauma is particularly dangerous due to its potential link to progressive neurological deterioration [3]. However, while neurodegenerative diseases such as chronic traumatic encephalopathy (CTE) can be suspected during life, they can only be confirmed postmortem [4], making them difficult to study.

TBI pathology is often categorized into primary and secondary injuries, the primary being the acute phase of injury that causes axonal shearing leading to hemorrhage and contusions, while the secondary injury is the result of molecular mechanisms involving cell death and tissue degeneration. Dying and damaged cells release debris that triggers the surrounding microglia and astrocytes to mount an immune response resulting in inflammation [5]. In relation to neuronal death, neurofibrillary tangles (NFTs) form and accumulate in the superficial layers of the cerebral cortex and at the depths of sulci, resulting in axonal instability and impeded neuronal communication [6, 7]. Microscopic imaging of these pathologies using histology provides an indicator on the progression of the disease. In TBI cases without focal lesions, sequelae such as behavioral changes or macroscopic regional brain shrinkage only appear at advanced stages of pathology [8], adding a layer of difficulty to diagnosis of mild and repetitive TBI.

Although the study of brain injury has advanced through animal models, mostly mice and rats, all neuroprotective therapies developed from rodent approaches have failed late-stage clinical trials, possibly due to the many morphofunctional differences between rodent and human brains [9]. Complementing rodent studies with alternative animal models with larger, gyrencephalic brains can increase our knowledge of the translational mechanics of neurodegenerative disease progression [10].

Many male animals of the artiodactyl order (mammals with even-toed hooves and cetaceans) perform head-to-head sexual displays during the reproductive season called the rut, usually sparring with their heads, horns, or antlers as a show of dominance in their social hierarchy. Among artiodactyls, caprines (sheep, goat, and muskox-type animals) exhibit the most extreme form of headbutting behavior. Muskox bulls (*Ovibos moschatus*) run towards each other and bash heads at peak speeds of 50 km/h. Bighorn sheep (*Ovis canadensis*) engage in forceful headbutting displays in mountainous terrain, both animals exerting forces of around 3000 N [11], aided by some of the proportionally largest horns in extant mammals. It is a common belief that headbutting animals like bighorn sheep are mostly unscathed after headbutting; however, this claim has not been investigated empirically, either through behavioral measures, or anatomically.

The biomechanics literature specifically has embraced this assumption with the aim to create biomimicry materials from horns for products such as helmets [12–18]. However, the anatomical aspects of the modeled animals lack comprehensive analysis [19]. Horns are certainly an important factor in the absorption of shock, but they cannot be the only structures in play, as females with smaller horns and dehorned domestic bovids also headbutt without sustaining apparent injury [20].

Headbutting behavior is not uniform across species or sexes. Bighorn ewes headbutt at lower forces but engage up to four times more often than males [20], and muskox cows also headbutt on occasion, without engaging in the long, ritualistic rut behavior observed in the males (Jamie Luce, The Musk Ox Farm, personal communication). Finally, bighorn rams often strike each other on a fat pad between their horns and on the forehead, rather than directly on the horns during fights [21]. Field observations have described muskoxen as “acting dazed” [22] or even “bleeding from the nose and ears after the rut” [23], but these observations were not confirmed anatomically. Whether bovids sustain any damage from these encounters in the form of chronic or acute brain trauma remains untested at both the macroscopic and microscopic levels, and yet sheep are one of the preferred large-animal models for TBI and CTE [24–26].

In this study, we aim to determine if bighorn sheep and muskoxen naturally sustain TBIs despite their large horns and thick skulls. In that instance, we expect males to follow a pattern resembling CTE in humans, with tau-immunoreactive staining of NFTs and neurites, especially in the superficial neocortical layers and in the depths of the sulci, coupled with activated microglia and astrocytosis. Macroscopic signs of TBI would only be expected to develop in the latest stages of neurodegeneration [27]. Understanding how bovids survive high-force head impacts can address the anatomical and physiological knowledge gaps with regards to current large animal TBI models, as well as providing further insight on the life history of these animals. Moreover, comparing TBI development between bovids and humans could lead to a better understanding of brain injury progression and treatment overall.

## Materials and methods

### Specimens

A total of nine brain specimens were used in this study: three from muskoxen, four from bighorn sheep, one from a human with late-stage Alzheimer’s disease (AD), and one from a human with CTE, the latter two used as positive controls. The brains of three wild adult muskoxen were collected during a field expedition to Ittoqqortoormiit, Greenland by TMW and all ages were estimated from tooth wear. During the time of brain recovery, the ambient temperature remained always below 7 °C, mitigating tissue deterioration. The male muskox (older adult, collected in summer 2019) was shot in the neck directly after a goring injury to the flank sustained while headbutting another male muskox. One female muskox was shot in the top of the head (middle-aged adult, collected in summer 2018) and the second female muskox was shot in the neck (very old adult, collected in summer 2018), for subsistence hunting (Table 1). The male muskox’s skull was approximately 4 cm thick in the frontal region (including sinuses), and 2 cm thick in the parietal cortex. The four bighorn sheep brains were collected as follows. The brain of an adult male bighorn sheep (Bighorn 1, five years old, collected in winter 2020) was acquired from Colorado Parks and Wildlife from a captive research herd after it was humanely euthanized (darted with NalMedA for sedation, euthanized with 20 ml Euthasol IV) due to a leg fracture. Formalin was injected around the brainstem and into the carotid artery for preservation before shipping. The male bighorn sheep’s skull was approximately 3 cm thick in the frontal region (including sinuses) and 1.5 cm thick in the parietal region. The brain of one wild adult female bighorn sheep (Bighorn 2, four years old, collected in winter 2020) was acquired from Utah Fish and Wildlife after euthanasia due to a *Mycoplasma* infection. The skull of the bighorn ewe was approximately 1.3 cm thick in the frontal region (including sinuses) and 0.8 cm thick in the parietal cortex. Two additional female bighorn sheep brains (Bighorn 3, five years old, collected in fall 1987; Bighorn 4, adult, collected in winter 2008) were archived in our collection and were provided by the Buffalo Zoo in 2003. After the muskoxen and sheep brains were removed from the skull, all samples were fixed in 10% formalin, either hours after death for the muskoxen, or within 36 h after death for the bighorn sheep. As a positive control for presence of phosphorylated tau, an archived human brain specimen with AD (male, 85 years old, Clinical Dementia Rating 3, Mini-Mental State Exam 11, Thal amyloid stage 4, Braak tangle stage V, postmortem interval 11 h, clinical diagnosis: severe cognitive impairment) [28] and one with CTE (male, 69 years old, postmortem interval 9 h, clinical history: repetitive athletic head injuries, post-mortem diagnosis of advanced CTE, moderate cerebrovascular disease—athero-arteriolosclerosis; moderate hypoxic–ischemic encephalopathy, severe postmortem autolysis) were used in this study.

**Table 1:** Bovid brain samples used in this study

ID	Sex	Age	Brain region	Origin
Bighorn 1	M	5 y.o	PFC	Captive
Bighorn 2	F	4 y.o	PFC	Wild
Bighorn 3	F	4 y.o	PFC	Zoo
Bighorn 4	F	Middle	PFC	Zoo
Old male muskox	M	Old	PFC Parietal	Wild
Middle-aged female muskox	F	Middle	PFC Parietal	Wild
Old female muskox	F	Old	PFC Parietal	Wild

Samples were taken from the prefrontal or parietal cortex of bighorn sheep or muskoxen of both sexes

*M* male, *F* female, *PFC* prefrontal cortex

### Magnetic resonance imaging (MRI)

MRI with superior soft tissue visualization was used to image the anatomical structure of the muskox and bighorn sheep brains. Coronal T2-weighed turbo spin-echo images were performed on a whole-body 7 Tesla (7 T) MRI scanner (Siemens Magnetom, Siemens Healthcare, Erlangen Germany) using a 1-channel transmit and 32-channel receive head coil (Nova Medical, Wilmington, Massachusetts) with the following parameters: repetition time (TR) 8000 ms, echo time (TE) 64 ms, number of sections 24, section thickness 1 mm, field-of-view 16 × 14 cm^2^, voxel size 0.5 × 0.5 × 1 mm^3^, scanning duration 6 min 20 s. Human tissue specimen imaging was performed in compliance with all institutional requirements.

### BLAST analysis

The tau-immunoreactive antibodies selected for this study were developed against human antigens. Their specificity in bighorn sheep and muskox tissues required validation to confirm their immunoreactivity. A basic local alignment tool (BLAST) compares protein sequences to sequence databases and calculates the statistical significance. The *MAPT* gene or protein sequence for tau was not available in bighorn sheep or muskoxen, therefore BLAST was applied to the predicted proteome of the domestic sheep (*Ovis aries*), the closest related species with MAPT sequence available on NCBI. Protein isoforms with the closest sequence homology and molecular weight to the requested sequence were ranked in terms of degree of identity in percent and *E* value. Homology values over 95% are considered acceptable, values over 85% are considered moderate.

### Immunohistochemistry and histochemical staining

**Fig. 1:**
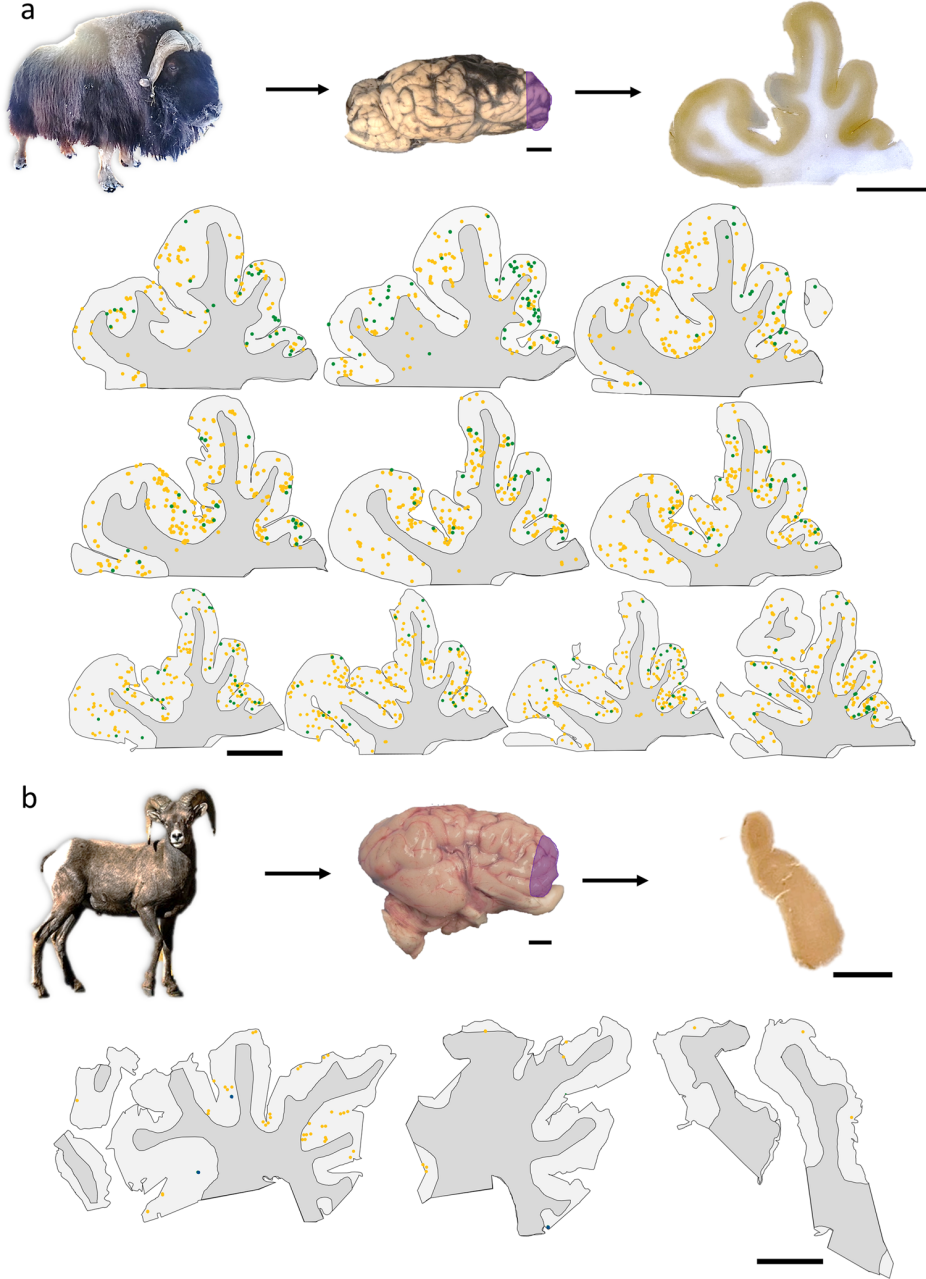
Sampling site and serial sections in bovid brains. **a** The brains were extracted from three muskoxen. Here, a block from the prefrontal cortex of the old male muskox is represented. Sections were cut at 50 µm-thickness from a block in the prefrontal cortex of the right hemisphere (purple shading). In serial sections each 500 µm apart, tau pathologies were counted exhaustively after pSer202 tau immunostaining. Neuropil threads are represented by yellow points and neuritic thread clusters by green points. Note the difference in shape between the sections with progression through the block. Muskox image courtesy of the Musk Ox Farm, AK. **b** Brain sections from the male bighorn sheep were pSer396/Ser404 tau-immunostained and tau pathologies were counted exhaustively. Neuropil threads are represented by yellow points and neurons are represented by blue points. No neuritic thread clusters were found in the bighorn sheep. Bighorn sheep image source: rawpixel.com. Scale bar = 1 cm

Brain tissue from the human subjects was sampled from Brodmann area 10. In the bovids, tissue was taken from the anterior region of the prefrontal cortex of the right hemisphere (Fig. 1), and additionally from the parietal cortex of the right hemisphere for the three muskoxen. Each block was cut into 50 µm-thick sections on a vibratome (Leica VT1000S) and stored in phosphate-buffered saline (PBS, pH 7.0) solution with 0.1% sodium azide. Multiple phosphorylated tau antibodies were tested in this study, as no protocols existed for these bovid species, making it uncertain which epitopes were present in each. Antibodies recognizing ionized calcium-binding adaptor molecule-1 (Iba1) and glial fibrillary acidic protein (GFAP) were used to investigate microglial and astrocytic morphology, respectively. Three antibodies were tested to detect the presence of phosphorylated tau protein (p-tau). Anti-CP13 is a p-tau antibody clone that binds an epitope around Serine 202 (pSer202 tau), the AT8 clone recognizes epitopes around Serine 202 and Threonine 205 (pSer205/Thr205 tau), and the PHF-1 clone was raised against epitopes including Serine 396 and Serine 404 (pSer396/Ser404 tau). Both CP13 and PHF-1 antibody clones were generously supplied by the Davies laboratory (Feinstein Institute for Medical Research, Northwell Health, Manhasset, New York, USA). Anti-neurofilament clone SMI-312 detects medium- and heavy-chain phosphorylated neurofilament proteins (pNFP) and was used to investigate axonal damage. Anti-denatured myelin basic protein (dMBP) was applied to assess possible demyelination. The MOAB-2 antibody clone was applied against amyloid beta protein (Aβ), and anti-TDP43 clone binds an epitope around phosphorylated Serine 409 and Serine 410 (pSer409/410) to detect pathological proteins. Anti-collagen IV was applied to highlight blood vessel morphology. Additional details on antibodies are reported in Table 2. Sections were stained either as single instances or, in the case of the muskoxen, as series of ten sections, each 500 µm apart for stereological quantification. Primary antibody controls were performed by omitting the primary antibody and assessed that the secondary antibody did not generate non-specific staining (supplementary figures S1 and S2, provided as an online resource).

**Table 2:** List of antibodies used in this study

Antibody	Full name	RRID	Company	Product number	Target	Dilution	AD human	CTE human	Muskox	Bighorn
AT8		AB_223647	ThermoFisher	MN1020	Phospho tau, Serine 202, Threonine 205	1:500	✓	✓	✓	✓
Collagen IV		AB_445160	Abcam	AB6586	Basement membrane of blood vessels	1:300	✓	✓	✗	✗
CP13		AB_2314223	Davies lab		Phospho tau, Serine 202	1:1000	✓	✓	✓	Weak
dMBP	Denatured myelin basic protein	AB_2140351	Sigma	AB5864	Denatured myelin	1:500	Weak	Weak	Weak	Weak
GFAP	Glial fibrillary acidic protein	AB_880202	Abcam	68428	Astrocytes	1:1000	✓	✓	✓	✓
Iba-1	Ionized calcium-binding adaptor molecule-1	AB_839504	Wako	019–19741	Microglia	1:500	✓	✓	✓	✓
MOAB-2	Amyloid beta antibody	AB_2895168	MilliporeSigma	MABN254	Amyloid beta	1:500	✓	✓	✗	✗
PHF-1		AB_2315150	Davies lab		Phospho tau, Serine 396, Serine 404	1:500	✓	✓	✓	Weak
SMI-312	Anti-phosphorylated neurofilament	AB_2566782	BioLegend	837904	Phosphorylated neurofilaments M and H in axons	1:1000	✓	✓	✓	✓
TDP43	Anti-TAR DNA-binding protein 43	AB_2750118	BioLegend	829901	Phosphorylated TDP43 protein, Serine 409, Serine 410	1:200	Weak	Weak	✗	✗

All washes were performed with either PBS (Iba1, GFAP, dMBP, collagen IV) or Tris-buffered saline (TBS, pH 7.0) (AT8, CP13, PHF1, TDP43, MOAB-2, SMI-312) at room temperature on a shaker at 80 rpm for five minutes each. Antigen retrieval for Iba1 was performed by submerging free-floating tissue sections in a 10 mM EDTA solution (pH 8.0) in closed 15-ml tubes. The tubes were immersed in a water bath at 80 °C for 10 min. For GFAP, AT8, MOAB-2, dMBP, and SMI-312, antigen retrieval was performed by submerging free-floating tissue sections in citrate buffer (pH 6.0), then boiled at 100 °C for 10 min, followed by 5 min of cooling down. Antigen retrieval for collagen IV was performed by submerging sections in a 0.5 M acetic acid and 10 mg/ml pepsin solution at 37 °C for 8 min.

After antigen retrieval, sections were transferred to 12-well plates using a glass hook and washed three times. Sections were then incubated with 0.3% hydrogen peroxide and 0.3% Triton X-100 in buffer for 30 min at room temperature, to inhibit endogenous peroxidase activity. The sections were then washed four times and blocked in buffer with 5% normal donkey serum (017000121, JacksonImmuno, West Grove, PA, USA) or normal goat serum (for TDP43 and collagen IV. 1002635372, Sigma, St. Louis, MD, USA) for 1 h, followed by an incubation in primary antibody (Iba1, GFAP, CP13, AT8, PHF1, MOAB-2, TDP43, SMI-312, dMBP, or collagen IV) in buffer with 5% normal donkey serum and 0.3% Triton X-100, at 4 °C overnight (Iba1, GFAP, AT8, MOAB-2, dMBP, SMI-312, collagen IV) or for 64 h (CP13, PHF1, TDP43); the control sections were incubated in buffer. After primary antibody incubation, sections were washed three times in buffer and 0.3% Triton X-100, then incubated with the appropriate secondary antibody (biotinylated donkey anti-rabbit, secondary antibody, 1:1000, 715065152, JacksonImmuno; biotinylated donkey anti-mouse, secondary antibody, 1:1000, 715065150, JacksonImmuno, biotinylated goat anti-rat secondary antibody 1:1000, BA9400, Vector laboratories, Burlingame, CA, USA) in a 5% normal donkey or goat serum and 0.3% Triton X-100 solution in buffer at room temperature for one hour (Iba1, GFAP, collagen IV, SMI-312) or two hours (AT8, CP13, PHF1, TDP43, MOAB-2, dMBP). After incubation with the secondary antibody, the sections were washed in buffer four times, then incubated with avidin–biotin solution (according to the manufacturer’s instructions, Vectastain ABC kit, Vector Laboratories, Burlingame, CA, USA) for one hour (Iba1, GFAP, MOAB-2, dMBP, collagen IV, SMI-312) or two hours (AT8, CP13, PHF1, TDP43) at room temperature. The sections were then washed four times, followed by an incubation with DAB peroxidase substrate (Vector Laboratories, according to the manufacturer’s instructions, DAB kit SK-4100) to reveal immunostaining. Samples were then mounted on gelatin-coated slides and left to dry overnight. After drying, the samples were counterstained with cresyl violet, dehydrated through an ethanol gradient, and coverslipped with DPX mounting medium.

Luxol Fast-Blue was applied to observe demyelination as follows. Mounted sections were incubated in 0.1% Luxol Fast-Blue solution for one hour at 56 °C, then rinsed in distilled water. Sections were then differentiated in a 0.05% lithium carbonate solution for 30 s, followed by 70% ethanol for 30 s. Sections were rinsed in distilled water and checked microscopically for differentiation. Finally, slides were rinsed and differentiated in 95% ethanol then coverslipped as above.

### Immunofluorescence

The blocking solution was increased from 5 to 10% normal donkey serum and primary antibody incubation was performed as in the immunohistochemistry protocol for both the CP13 and GFAP antibodies. After primary antibody incubation, sections were washed four times, protected from light, and incubated in biotinylated donkey anti-mouse antibody (1:1000, 715065150, JacksonImmuno) and anti-rabbit-AlexaFluor 488 (1:1000, A31570, ThermoFisher Scientific), followed by streptavidin-AlexaFluor 555 (1:500, A21206, ThermoFisher Scientific) in 10% normal donkey serum and 0.1% Triton X-100 in TBS at room temperature for two hours. The sections were washed four times and then incubated in 10% normal donkey serum and 0.1% Triton X-100 in TBS at room temperature for two hours. Sections were then washed four more times, mounted on SuperFrost slides and dried for one hour at 50 °C. Wells were drawn around the sections with an ImmEdge pen and sections were washed for 10 min. Sections were then incubated with TrueBlack (diluted 20 × in 70% ethanol) for 30 s each to reduce autofluorescence, then washed four times. The sections were then incubated with 4′,6-diamino-2-phenylindole dihydrochloride (DAPI, 250 ng/ml) for ten minutes in a humid chamber to stain cell nuclei and then washed a final time, after which they were mounted under Vectashield (H1000, Vector Laboratories) and coverslipped (24 × 50 mm No.1.5 ThermoFisher Scientific).

### Microscopy and stereology

Brightfield microscopy images were taken on an Axiophot brightfield microscope (Carl Zeiss Microscopy, Jena, Germany), with a 10×/0.32 Plan-Apochromat objective using StereoInvestigator (version 11.03, MBF Bioscience, Williston, VT, USA). Fluorescence images were taken on a CLSM 780 confocal microscope (Carl Zeiss Microscopy), using a 20×/0.8 DICII objective and DPSS 561-10 diode and Argon lasers at excitation wavelengths of 461, 555, and 488 nm. Confocal stacks in layers II and III of the cerebral cortex were imaged at 512 × 512 pixel resolution with a Z-step of 1 µm and a pinhole setting of 1 Airy unit for the red wavelength and optimal settings for gain and contrast. Images are presented as maximum intensity projections of the Z-stack using ZenBlue (version 3.3, Carl Zeiss Microscopy).

Stereological quantification of tau immunostaining was performed using the optical fractionator workflow probe in StereoInvestigator (magnification × 10, counting frame size 700 × 700 µm, SRS grid layout at 100% of the region of interest, optical dissector height 11 µm with 2 µm of top and bottom guard zones, manual focus), on each muskox specimen in a series of 10 sections, each separated by 500 µm, and counted exhaustively. Cortical layers were manually contoured into layers I, II, III, IV–VI, and white matter. In each layer, different markers were placed for tau-immunoreactive neuropil threads (axonal or dendritic filaments composed of abnormally phosphorylated microtubule-associated tau protein), neuritic thread clusters (circular dense cluster of neuritic threads), and neurons (neuronal cells with tau-immunoreactive inclusions in the cytoplasm). Section contours were aligned manually and the coordinates were exported to create individual and combined heatmaps of tau-immunoreactive neuropil density distribution in Rstudio [29] using the ggplot2() package [30]. Annotated R code and raw data is available on GitHub (https://github.com/NLAckermans/Ackermans2022BovidTBI.git).

To quantify tau-immunoreactive pathology accumulation in the sulci as opposed to gyri in the muskox specimens, sulcal depths were delimitated as 1/3 of the sulcus as in [31] and pSer202 tau-immunoreactive markers in each region were quantified in StereoInvestigator using the same specifications as above. To quantify pSer202 tau-immunoreactive pathology around blood vessels in the muskoxen as compared to the CTE human control, one section from each individual was subjected to exhaustive counting of vessels larger than 30 µm in diameter at 2.5 × magnification with StereoInvestigator’s Optical Fractionator probe. The percentage of vessels immunostained with pSer202 tau located within 100 µm of the edge of the vessel and the average distance from the edge of the vessel were calculated and reported.

## Results

**Fig. 2:**
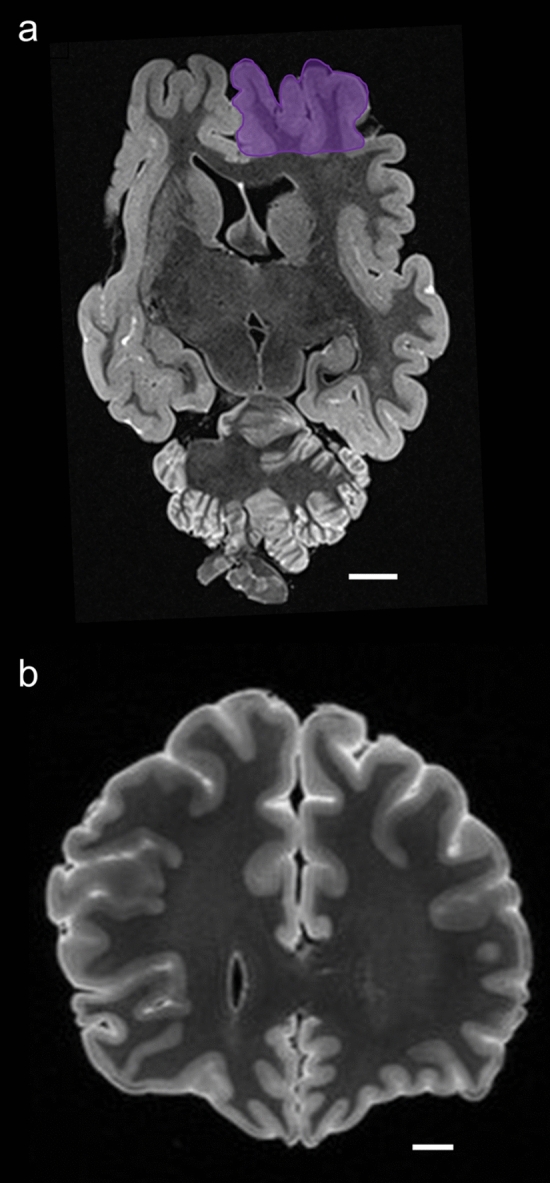
Magnetic resonance imaging (MRI) coronal scans of a muskox and a human brain. **a** Male muskox brain ex vivo, before sampling; the right prefrontal cortex is shaded in purple. Deformation and damage to the cerebellum occurred postmortem. Image obtained by coronal T2-weighted turbo spin-echo images obtained from an ex vivo old male muskox brain at 0.57 mm in-plane resolution and 1 mm slice thickness using a 7 T MRI scanner. **b** Human brain with a history of TBI, frame taken through the human prefrontal cortex at the level of the corpus callosum. Image obtained by ex vivo coronal 3D T2-weighted fluid attenuated inversion recovery (FLAIR) turbo spin-echo image acquired at 3 T at 0.63 mm isotropic resolution. Scale bar = 1 cm

None of the specimens showed any external brain trauma and the MRI scans were reviewed by a neuroradiologist for internal signs of TBI pathology such as shrinkage, acute trauma, or microhemorrhages in any of the bovid brains (Fig. 2). Such alterations were not observed in our specimens. The male muskox brain only showed ex vivo artifacts of deformation and damage, due to field conditions and storage, but none were related to TBI (Fig. 2A), in comparison to a control specimen of a human brain with severe TBI (Fig. 2B).

BLAST protein analysis for human MAP-tau against the estimated domestic sheep genome indicated the presence of protein with a comparable amino acid sequence in this species. The homology degree between the human (accession number P10636) and domestic sheep X1 (XP_042112063.1) and X4 (XP_027830174.1) isoforms was 80% and 87%, respectively.

**Fig. 3:**
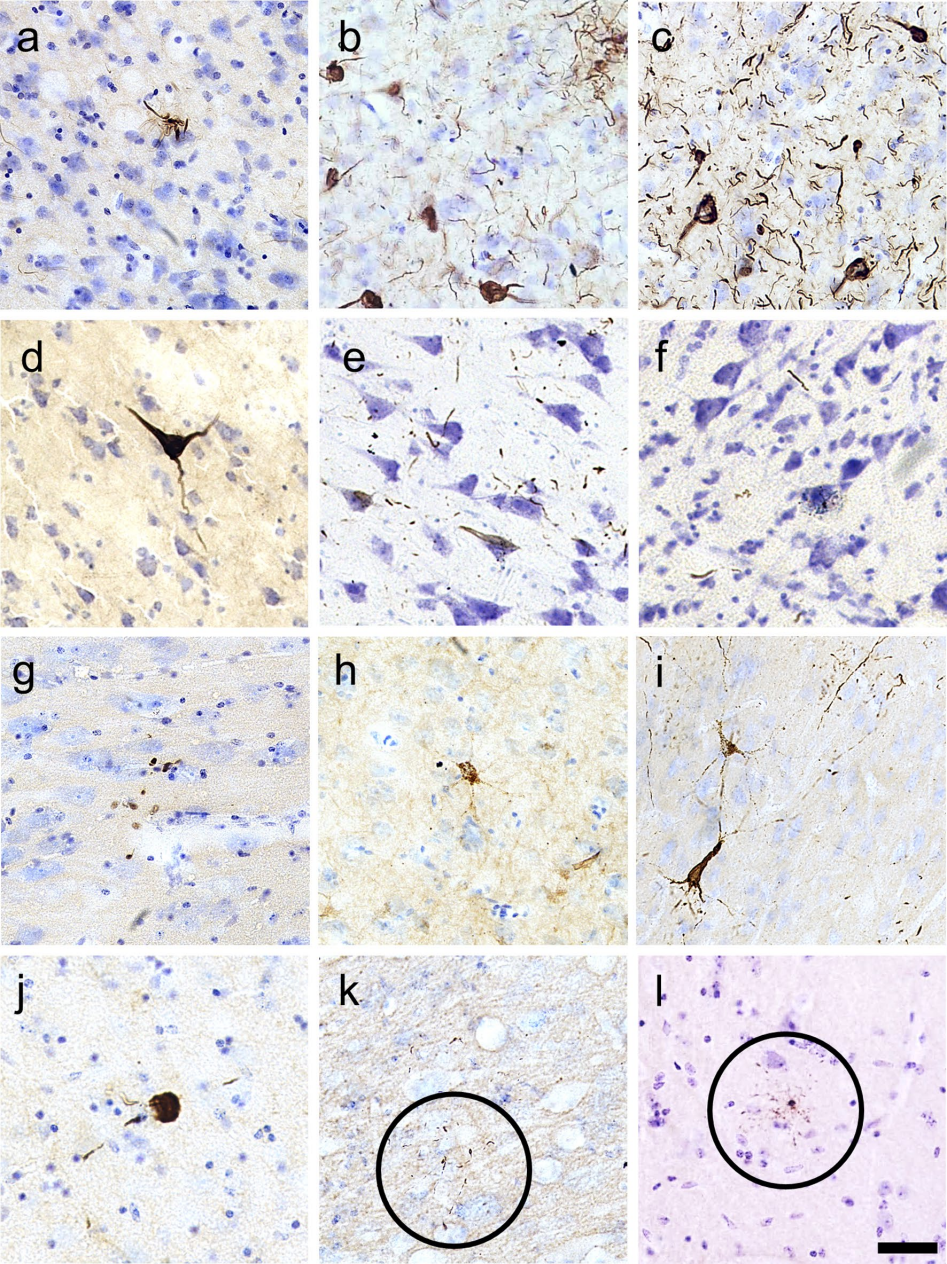
Micrographs of abnormally phosphorylated tau immunostained using different antibodies in brains of human with late-stage Alzheimer’s disease (**a**–**c**), human with CTE (**d**–**f**), old male muskox (**g**–**i**), and a male bighorn sheep (**j**–**l**). Samples were taken from the prefrontal cortex and immunostained for pSer396/Ser404 tau (**a**, **d**, **g**, **j**), pSer202/Thr205 tau (**b**, **e**, **h**, **k**), and pSer202 tau (**c**, **f**, **i**, **l**). In the human AD specimen, pSer396/Ser404 tau-immunoreactive (**a**) neuropil threads, (**b**) pSer202/Thr205 tau-immunostained neuritic thread clusters, neuropil threads, and neurons, and (**c**) pSer202 tau-immunostained neurons and neuropil threads. In the human CTE specimen, pSer396/Ser404 tau-immunoreactive (**d**) neuron, (**e**) pSer202/Thr205 tau-immunoreactive neurons and neuropil threads, and (**f**) pSer202 tau-immunoreactive neuritic threads. In the old male muskox pSer396/Ser404 tau-immunoreactive (**g**) neuropil threads, (**h**) a pSer202/Thr205 tau-immunoreactive neuron, and (**i**) pSer202 tau-immunoreactive neuritic thread clusters, neuropil threads, and neurons in layer II near a blood vessel. In the male bighorn sheep one of the only pSer396/Ser404 tau-immunoreactive structures (**j**) a neuron and associated neuropil threads; (**k**) pSer202/Thr205 tau-immunoreactive neuropil threads, circled; (**l**) pSer202 tau-immunoreactive neuropil threads, circled. Scale bar = 50 µm. Controls in Fig. S1

**Fig. 4:**
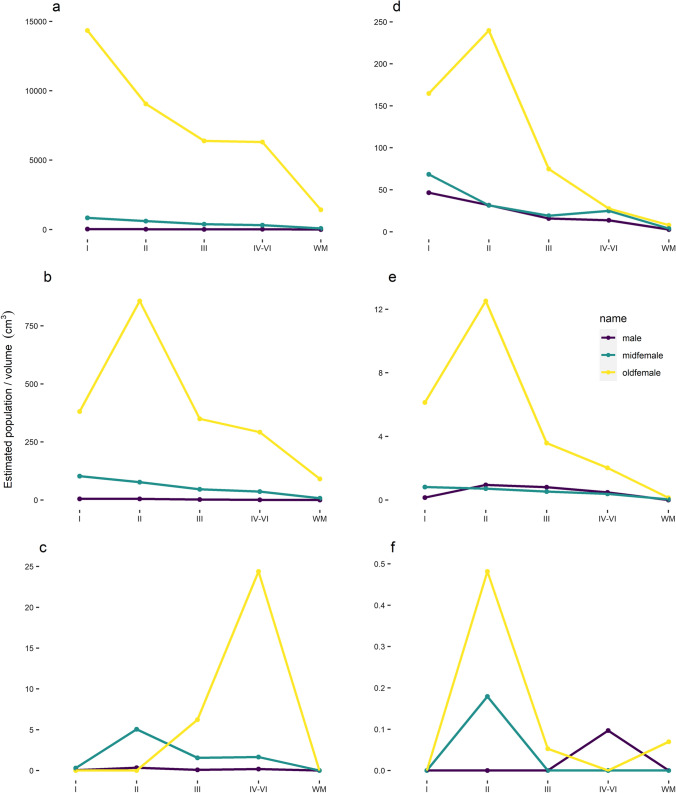
Distribution of pSer202 tau-immunoreactive structures among neocortical layers in the muskox. Brain samples from the prefrontal (**a**–**c**) and parietal cortex (**d**–**f**) of an old male, middle-aged female, and old female muskox. Sections were pSer202 tau immunostained, and an exhaustive count of tau-immunoreactive (**a**, **d**) neuropil threads, (**b**, **e**) neuritic thread clusters, and (**c**, **f**) neurons was performed using StereoInvestigator on ten slices, each 500 µm apart. Estimated population was corrected for volume per brain layer (cm^3^). Note the scale difference for each graph

**Fig. 5:**
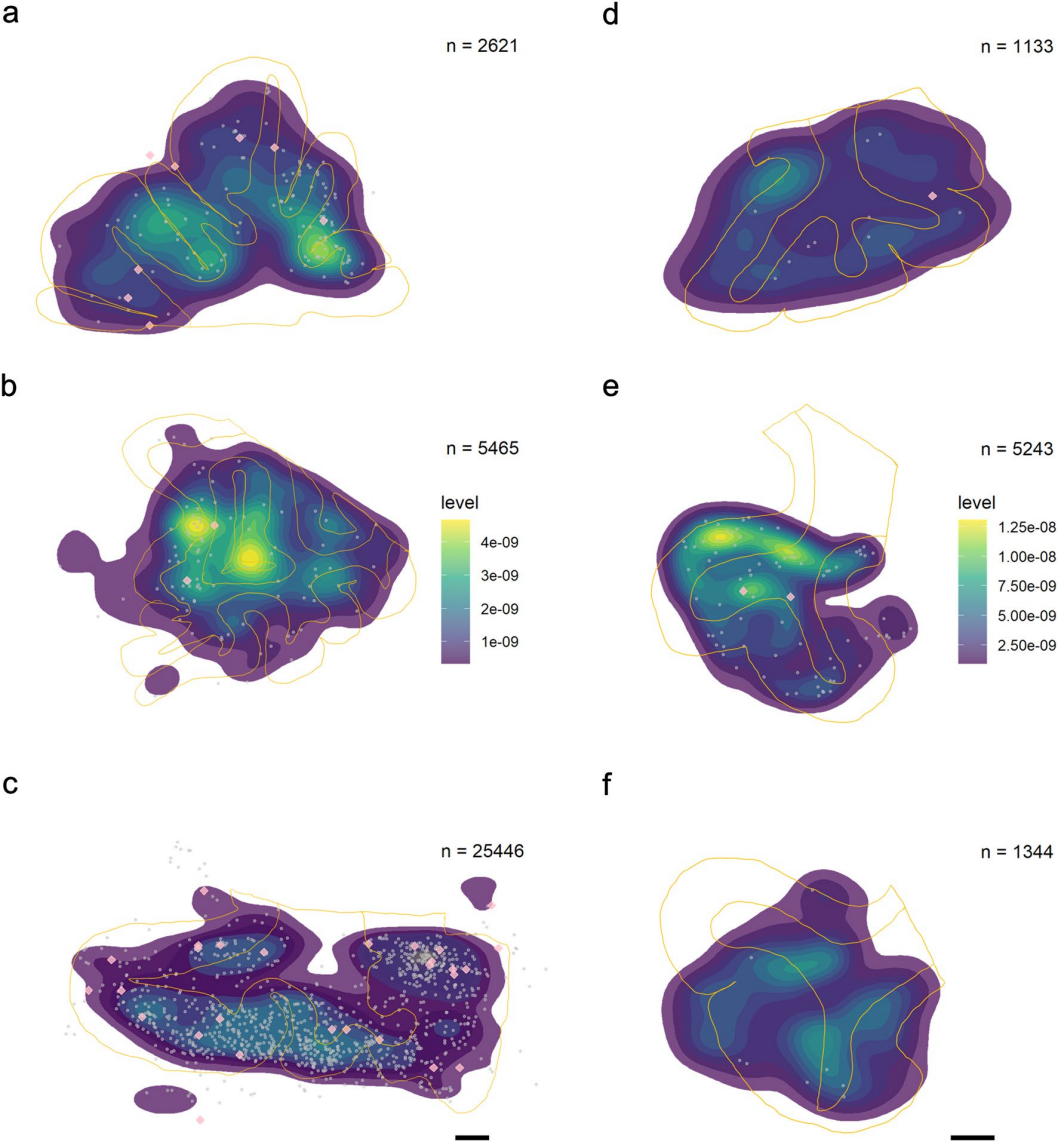
Distribution of pSer202 tau-immunoreactive structures recorded in neocortical sections of muskox brain. Heatmaps represent the total distribution (not the estimated population) of pSer202 tau-immunoreactive neuropil threads throughout 500 µm of brain tissue in 10 sections. Tissue is from the prefrontal cortex in **a** the male muskox, **b** the middle-aged female muskox, and **c** the old female muskox, and from the parietal cortex of the right hemisphere in the **d** male muskox, **e** middle-aged female, and **f** old female muskox. Yellow lines mark the average outline of white and grey matter in each individual throughout the ten slides. Grey points indicate pSer202 tau-immunoreactive neuritic thread clusters and pink diamonds represent pSer202 tau-immunoreactive neurons. Note that the highest densities of neurites are at the base of the sulci and that higher neuropil thread counts in **c** does not necessarily equate higher density. The highest densities of neuropil threads are in yellow, note the different scale in the two brain regions in **a**–**c** and **d**–**f**, calculated separately because of the difference in scale, which would have hidden density patterns. Scale bar = 1 cm

**Fig. 6:**
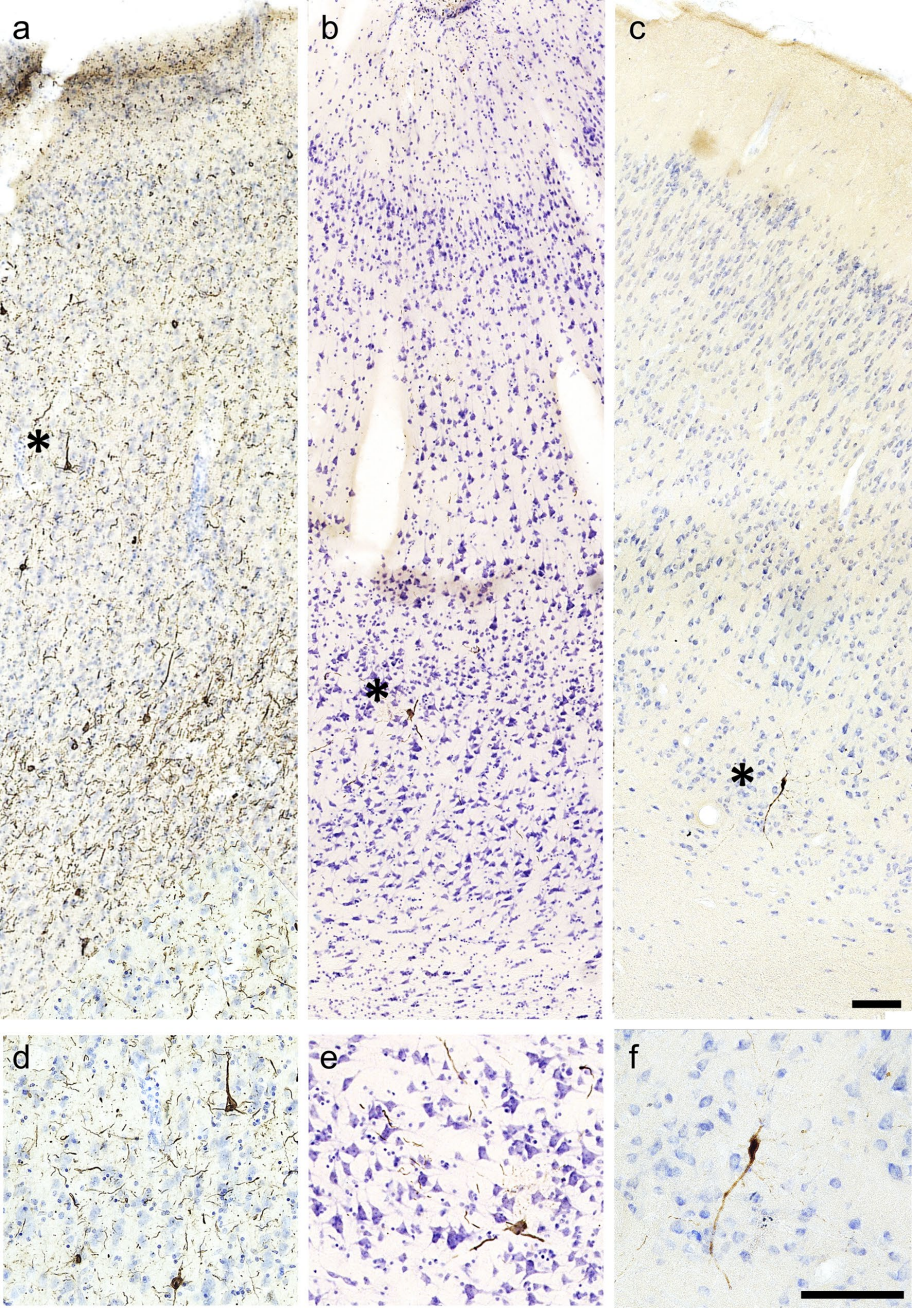
Tau-containing neurons in **a** human late-stage Alzheimer’s disease and **b** a human CTE case and **c** muskox brain. All sections were taken from the prefrontal cortex and pSer202 tau immunostained. Asterisks represent the magnified areas shown in **d**–**f**. Note the accumulation of pSer202 tau-immunoreactive structures in the AD human are well visible whereas in the CTE case, it is more sparse, similar to the muskox. Scale bar = 100 µm. No pSer202 tau-immunoreactive lesions appeared in a clinically healthy human case

All three p-tau antibodies (anti-pSer202 tau, anti-pSer202/Thr205 tau, and anti-pSer396/Ser404 tau) were applied to all three species (Table 2). All showed detectable signal in human and muskox, with anti-pSer202 tau specifically providing the most robust signal in muskox (Figs. 1A, 3A-I, controls in Fig. S1C); however, these antibodies rarely showed detectable signal in the bighorn sheep (Figs. 1B, 3J–L, controls in Fig. S1G–I). In the muskoxen, estimated population counts corrected for volume (Table 3) and exhaustive counts (Table S1) revealed pSer202 tau-immunoreactive structures in all the muskox specimens (Figs. 4, 5). pSer202-immunoreactive tau was present in neuropil threads, neuritic thread clusters, and neurons of the prefrontal and parietal cortex. The coefficient of error for tau-immunoreactive population estimates was in an acceptable range (< 15%) for all counts except neurons containing tau, in most cases due to their overall rarity in the sample (Table S2). In the bighorn sheep samples, tau-immunoreactive lesions mostly presented as pSer396/Ser404 tau-immunoreactive neuropil threads in the male bighorn, which were all in the grey matter and showed one grouping at the bottom of a sulcus (Fig. 1B, 3J). The positive control human CTE case showed a high number of tau-immunoreactive structures, especially neurons, heterogeneously clustered throughout the sample, especially around sulci and in the superficial cortical layers (Figs. 3D–F, 6B, E). The human AD case showed a much higher density of homogeneous tau immunoreactivity (Figs. 3A–C, 6A, D). Exhaustive stereological counting was, therefore, not performed in either the bighorn sheep or the human control samples.

**Table 3:** Density of different pSer202 tau-immunoreactive structures in muskoxen prefrontal and parietal cortical layers

Individual	Block	Region	Neuropil	NTC	Neuron
Male	PFC	I	26.38	5.05	0.04
		II	24.15	4.66	0.35
		III	12.81	2.01	0.09
		IV–VI	15.26	0.83	0.19
		WM	2.58	0.14	0.02
	Parietal	I	46.56	0.16	0
		II	31.58	0.95	0
		III	15.74	0.81	0
		IV–VI	13.72	0.48	0.1
		WM	2.67	0	0
Middle-aged female	PFC	I	838.06	102.55	0.3
		II	606.09	76.88	5.06
		III	386.74	46.31	1.56
		IV–VI	310.93	36.71	1.66
		WM	84.21	7.8	0
	Parietal	I	68.31	0.82	0
		II	31.34	0.72	0.18
		III	19.11	0.54	0
		IV–VI	24.9	0.39	0
		WM	3.81	0.04	0
Old female	PFC	I	14,345.69	380.7	0
		II	9049.63	856.97	0
		III	6385.27	349.36	6.24
		IV–VI	6306.02	292.55	24.38
		WM	1428.71	91	0
	Parietal	I	164.73	6.13	0
		II	239.71	12.51	0.48
		III	74.79	3.58	0.05
		IV–VI	27.55	2.02	0
		WM	7.81	0.14	0.07

Brain samples were taken from the prefrontal and parietal cortex and immunostained for pSer202 tau. An exhaustive count of pSer202 tau-immunoreactive neuropil threads, neuritic thread clusters, and neurons was performed using StereoInvestigator on ten slices, each 500 µm apart and adjusted for volume (cm^3^) of each layer. Exhaustive counts are reported in Table S1. Data represent estimated population/cm^3^ and are adjusted by volume

*WM* white matter, *PFC* prefrontal cortex, *NTC* neuritic thread clusters

### Tau-immunoreactive neuropil thread quantification

Density of pSer202 tau-immunoreactive structures was calculated by dividing exhaustive counts by sample volume. Estimated population counts in muskoxen prefrontal cortex and parietal cortex indicated that neuropil threads were the most common structure by far in all individuals. At their highest density, neuropil threads were twice as numerous as neuritic thread clusters in the prefrontal cortex and 60 times more numerous than pSer202 tau-immunoreactive neurons. In the prefrontal cortex of the old female muskox pSer202 tau-immunoreactive neuropil thread density was over 500 times more numerous than in the old male and around 16 times more numerous than in the middle-aged female muskox (Table 3). In the parietal cortex, the old female muskox still showed the highest density of pSer202 tau-immunoreactive neuropil threads with five times more than the old male and three times more than the middle-aged female muskox (Table 3). In the prefrontal cortex, neuropil thread density decreased with layer depth in all three specimens (Fig. 4A). Whereas in the parietal cortex, the old female showed the highest density in layer II, the old male and middle-aged female showed decreasing densities with layer depth (Fig. 4D). Depending on the individual and cortical layer, the prefrontal cortex showed about a sixfold density increase as compared to the parietal cortex. Distribution heatmaps based on neuropil thread density show the highest concentrations in the superficial layers and at the bottom of sulci for all specimens and both brain regions (Fig. 5), the latter of which was confirmed by stereological quantification (Table 4). Despite the highest density of pSer202 tau-immunoreactive neuropil threads being in the old female muskox, the other two specimens showed higher neuropil densities in the sulci, especially in the prefrontal cortex, indicating a more numerous but also more even distribution of tau-immunoreactive neuropils in the old female (Fig. 5). Overall, there were very few pSer202 tau-immunoreactive neuropil threads in the white matter (Figs. 4, 5).

**Table 4:** Density of pSer202 tau-immunoreactive structures per volume in sulcal vs gyral regions of the muskox cerebral cortex

Individual	Region	Neuropil	Neuropil sulcus	NTC	NTC sulcus	Neuron	Neuron sulcus
Male	PFC	6.86	23.23	0.88	3.10	0.07	0.13
	Parietal	10.54	25.99	0.22	1.18	0.03	NA
Middle-aged female	PFC	209.65	508.41	24.70	55.15	0.62	2.21
	Parietal	14.53	35.06	0.28	0.57	0.02	NA
Old female	PFC	34.49	115.16	2.10	3.72	0.01	0.19
	Parietal	63.12	125.29	3.18	3.46	0.07	NA

Brain samples were immunostained for anti-pSer202 tau. An exhaustive count of pSer202 tau-immunoreactive neuropil threads, neuritic thread clusters, and neurons was performed using StereoInvestigator on ten slices, each 500 µm apart and adjusted for volume (counts/mm^3^)

*NTC* neuritic thread cluster, *PFC* prefrontal cortex, *NA* no neurons counted

### Tau-immunoreactive neuritic thread cluster quantification

The density of pSer202 tau-immunoreactive neuritic thread clusters was eight times higher in the old female than in the middle-aged female muskox and about 20 times higher in the middle-aged female muskox than in the male (Table 3). The clusters were 5 to 100 times more frequent in the prefrontalthan in the parietal cortex depending on the individual. The middle-aged female muskox showed a pattern of decreasing density of neuritic thread clusters from the cortical surface to the deeper layers in both brain regions, while the male showed a similar pattern in the prefrontal cortex but a higher density in layer II of the parietal cortex. The old female muskox had the highest density of neuritic thread clusters in layer II in both brain regions with a more drastic pattern than the other two individuals (Fig. 4B, E). Overall, apart from being less numerous, neuritic thread clusters followed a similar distribution to the neuropil threads, especially in the parietal cortex, and were most concentrated in the superficial layers and at the base of the sulci (Fig. 5, Table 4).

### Tau-immunoreactive neuron quantification

Estimated and exhaustive counts both indicated pSer202 tau-immunoreactive neurons as relatively rare in all muskox specimens. However, they were five times more numerous in the old female than the middle-aged female and about 12 times denser in the middle-aged female than the old male muskox in the prefrontal cortex (Table 3). Although the low counts make estimates somewhat unreliable, tau-containing neurons were at their highest densities in layers IV–VI for the old female and layer II for the middle-aged female muskox, with very few pSer202 tau-immunoreactive neurons in the parietal cortex (Fig. 4C, F). Some grouping is visible at the base of a sulcus in the prefrontal cortex (Fig. 5) and slightly more tau-immunoreactive neurons clustered at the bottom of the sulci then elsewhere in the sample (Table 4). Overall, pSer202 tau-immunoreactive neurons were more numerous in the prefrontal than the parietal cortex, especially for female muskoxen (Tables 3, S1).

**Fig. 7:**
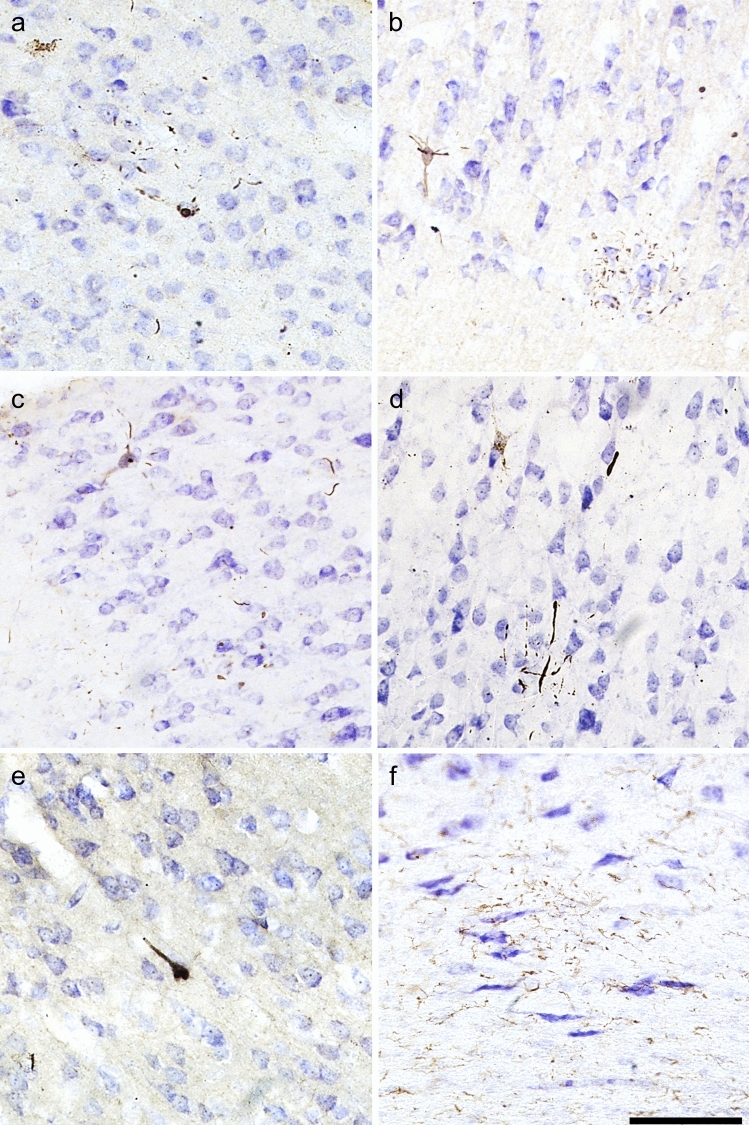
Photomicrographs of pathological structures in the parietal cortex of muskoxen. **a**–**c** pSer202 tau-immunoreactive pretangle-like features in pyramidal cells and neurites in the old female muskox. **d** pSer202/Thr205 tau-immunoreactive pretangle like features in a pyramidal cell and neurites in the old female muskox. **e** pSer396/Ser404 tau-immunoreactive NFT-like lesion in a layer III pyramidal cell in the old female muskox. **f** Iba1-immunoreactive microglial aggregation on the border of the white matter in the middle-aged muskox. Scale = 100 µm

Non-neuronal cell types were not immunostained by any of the three tau antibodies applied in the bovid specimens, as opposed to what is commonly seen in human CTE cases. Tangle-like structures were observed in the parietal cortex of the muskox brains and are shown in Fig. 7.

### Blood vessel association with p-tau pathology

**Fig. 8:**
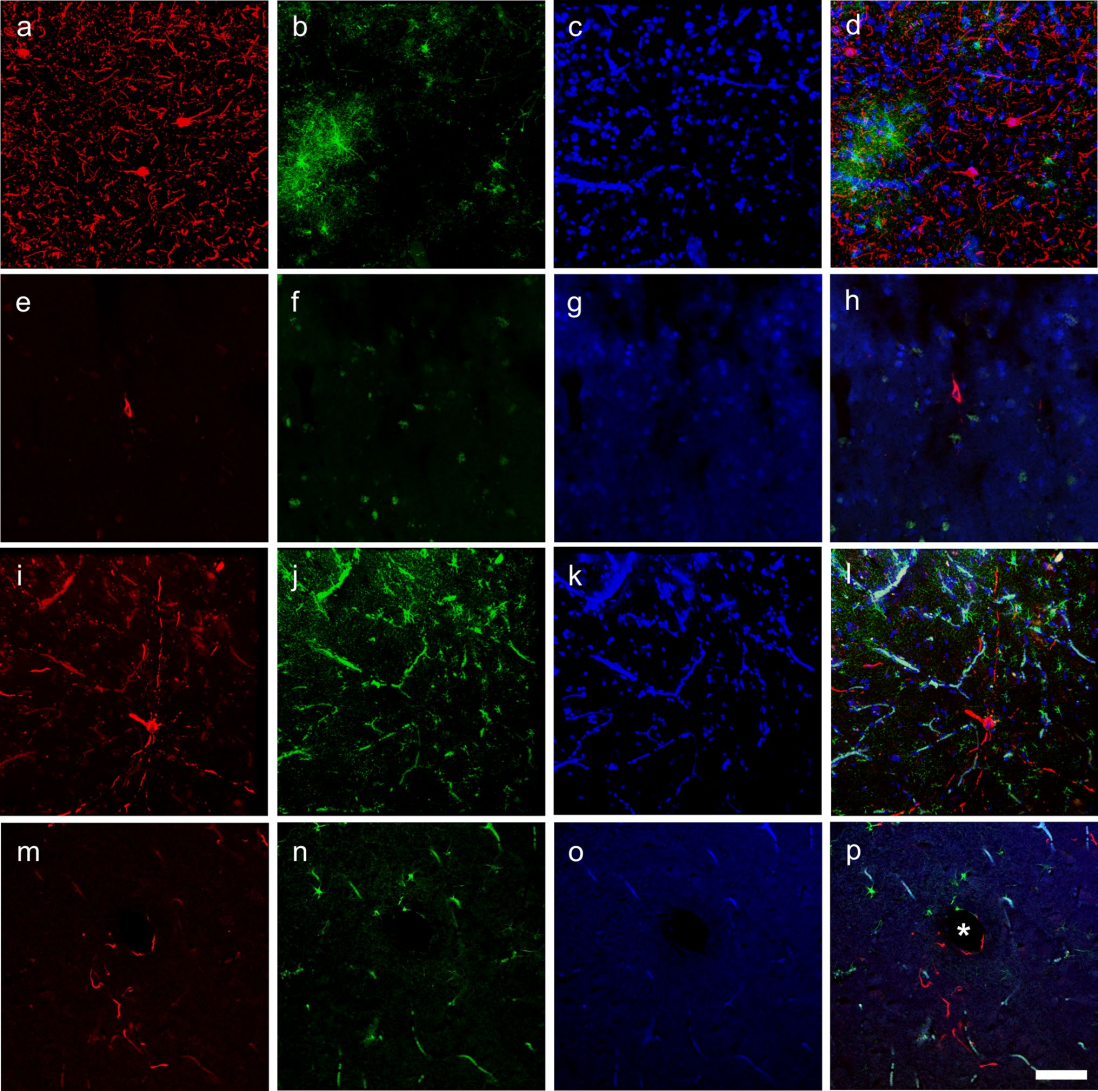
Immunofluorescence photomicrographs of pSer202 tau-immunoreactive structures and GFAP-immunoreactive astrocytes in human and muskox brain. In Brodmann area 10 of the human specimen with late-stage AD, **a** anti-pSer202 tau (red), **b** astrocytes are immunostained for GFAP (green), **c** nuclei are stained with DAPI (blue), and all three are combined in **d**. In the human specimen with CTE, tissue was immunostained with the same antibodies (**e**–**h**), as was the old male muskox prefrontal cortex (**i**–**l**) and the old female muskox parietal cortex tissue (**m**–**p**). In **d** note the activated astrocytes, in **l** and **p** the autofluorescence of blood vessels and in **l** astrocyte activation in the male muskox. Astrocytes and neuropil threads are encircling a blood vessel in the old female muskox represented by an asterisk (**p**). Scale bar = 50 µm

All three types of pSer202 tau-immunoreactive structures were found around blood vessels (Figs. 3I, 8P). When quantified in muskoxen, 4–20% of blood vessels were associated with these structures (Table 5). The old female muskox showed the highest percentage of blood vessels associated with pSer202 tau-immunoreactive structures, with an average distance from the blood vessel that was also smaller than in the other two cases at around 40 µm. As a comparison, the same quantification applied to a human CTE case revealed around 60% of blood vessels to be associated with these structures, at an average distance of around 30 µm.

**Table 5:** Quantitative analysis of blood vessel association with pSer202 tau-immunoreactive structures in muskox cerebral cortex

Individual	Total vessels	Total tau-associated vessels	% tau-associated vessels	Average vessel-tau distance (µm)
Old male	190	8	4.0	70.8
Middle-aged female	202	9	4.5	54.4
Old female	260	60	23.1	41.0
CTE human	94	58	61.7	29.9

### Glia

**Fig. 9:**
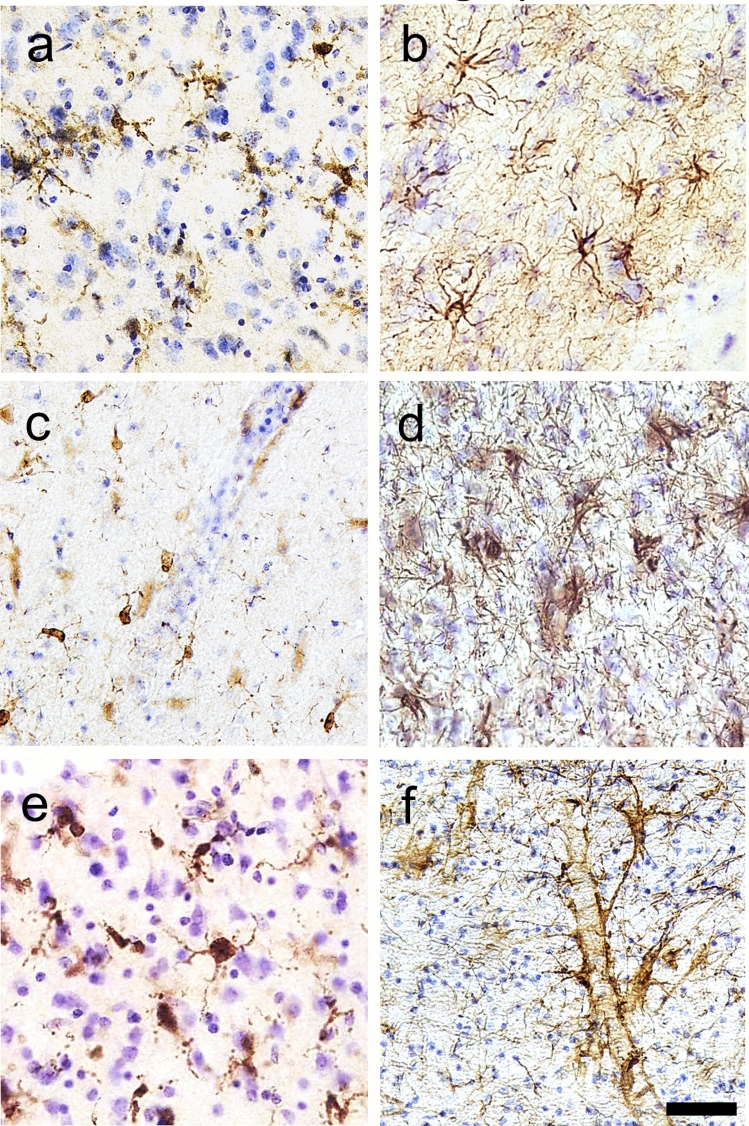
Photomicrographs of microglia (**a**, **c**, **e**) and astrocytes (**b**, **d**, **f**) in brains of human with late-stage AD (**a**, **b**), old male muskox (**c**, **d**), and a male bighorn sheep (**e**, **f**). Samples were taken from the prefrontal cortex and immunostained for Iba1 to detect microglia, or GFAP to detect astrocytes. Note astrocyte clumping in **b** but not in **f**. Note the astrocytic processes with endfeet around the blood vessel in **f**. Scale bar = 50 µm. Controls in Fig. S2

Microglia and astrocytes were labeled using antibodies Iba1 and GFAP in all species. In the AD and CTE human specimens microglia with abnormal morphology and astrocytosis were observed in association with neurodegenerative changes, but such changes were rarely visible in either bovid species in our study (Figs. 8L, 9). One microglial cluster was observed in the parietal cortex of the middle-aged female muskox (Fig. 7F). Furthermore, none of the muskoxen showed neurons or neurites immunoreactive for pSer202 tau in combination with activated astrocytes detected by GFAP (Fig. 8L, P), although this combination was apparent in the human AD specimen (Fig. 8D).

### Additional immunohistochemistry and staining

Luxol Fast-Blue was used to investigate demyelination, as dMBP did not produce immunoreactivity (Supplementary Fig. S3G-I). Luxol Fast-Blue staining showed no pathological changes (Fig. S4). In addition, anti-pSer409/410, which was present in the human sample did not reveal any pathology in the bovids (Supplementary Fig. S3J-L). Similarly, anti-Aβ revealed Aβ plaques in the CTE human specimen and anti-collagen IV revealed immunostained blood vessels in the human specimens but not in the bovids (Supplementary Fig. S3). The pNFP-immunostained axons were well visible in the muskox specimens, but no axonal damage was observed (Fig. S5).

## Discussion

This study assessed whether headbutting behavior in bovids is linked to TBI, mainly using tau-immunoreactive structures. We specifically chose bighorn sheep and muskoxen for this study as the most extreme representatives of their order. They headbutt at the highest forces in the animal kingdom and have evolved thick skulls and large headgear through sexual selection.

The MRI scans showed no macroscopic signs of TBI and susceptibility-weighted imaging showed no microhemorrhages in these animals. This was expected, as the skulls were intact in all specimens and brains showed no outer macroscopic signs of brain trauma, such as regional shrinkage, which only becomes evident in the late stages of neurodegeneration in humans [4]. Immunohistochemical p-tau immunostains on the bovid brains revealed a large amount of abnormally phosphorylated tau-immunoreactive neuropil threads, neurons, and neuritic thread clusters in the prefrontal cortex and the parietal cortex to a lesser extent. In the muskoxen, while anti-pSer202/Thr205 tau and anti-pSer396/Ser404 tau showed immunoreactivity, anti-pSer202 tau was most prevalent in all three structures and was, therefore, used for stereological quantification in both brain regions of the muskoxen. pSer202 tau-immunoreactive structures were concentrated in the superficial layers of the cerebral cortex, at the depths of the sulci, and occasionally around blood vessels. This distribution pattern was reminiscent of mild TBI or early-stage CTE cases, in American football players for example [32], and in other causes of repeated head trauma in humans [33]. This pattern differs from the tau distribution pattern in cases of AD in which the neocortical distribution of tau NFTs exhibits a well-defined bilaminar pattern [34–37].

Some pretangle-like structures with cytoplasmic tau immunoreactivity were observed in the muskoxen, mainly in the parietal cortex. Pretangles are an early form of NFT, representing a protective state of neuronal defense against abnormal tau in human neurodegeneration [38] and consistent with an early CTE diagnosis. Microglia and astrocyte morphology in our samples appeared relatively normal, apart from a few microglial aggregates observed in the middle-aged muskox. Glial activation and association with tau-immunoreactive neurons is diagnostic of human TBI [39] and potentially also CTE [40], but were not observed in our specimens. As perivascular tau can be indicative of CTE [41], we sought to quantify its severity in our specimens. Our results show that the old female muskox had a higher percentage of tau-immunoreactive-associated blood vessels than the other two muskoxen, closer to that measured in the human CTE case.

Our original hypothesis stated that if headbutting bovids sustained brain trauma at all, it would be most evident in the males, as they headbutt more often and at higher forces than the females. We also expected the oldest specimens to show the most pathology because of the cumulative effects of brain injury potentially acquired over years of repetitive headbutting. Our quantitative results highlighting immunoreactive structures with anti-pSer202 tau told a somewhat different story, with the old male muskox showing the lowest number of pSer202 tau-immunoreactive structures and the old female showing the highest by a factor of up to 500 in the case of neuropil threads. This seems to indicate a cumulative aspect of chronic TBI in the older individual and surprisingly, a much higher avoidance of TBI in the male than in the female specimens.

TBI caused by frequent and forceful headbutting in male muskoxen may be mitigated to a certain extent by their extreme anatomy, as a male muskox skull is on average 300% heavier than those of females [22]. Muskox bulls most likely show additional dimorphism in protective soft tissue structures like neck musculature, forehead fat pads, and meninges, as well as in postcranial and vertebral elements [22, 42]. The discrepancy in tau-immunoreactive densities between the two females could be caused by individual differences, as varying degrees of tau phosphorylation are also present in middle-aged humans with no behavioral differences [43]. Alternatively, social hierarchy may also have come into play, as new herd members of both sexes headbutt to establish dominance (Jamie Luce, The Muskox Farm, personal communication). In combination with these factors, age is likely the main contributing factor to the high tau-positivity in the old female, representative of accumulated chronic brain trauma in combination with age-related neurodegeneration, as indicated by the more evenly distributed neuropil threads and high occurrence of tau-immunoreactive structures in layer II.

High numbers of tau-immunoreactive neurites, neurons, and neuritic thread clusters are indicative of repetitive brain trauma in human cases [32, 33, 44, 45]. While early-stage CTE shows no gross abnormalities in most brains, perivascular NFT clusters, neuropil threads, and astrocytic tangles are found in the sulcal depths of the brain at the microscopic level [3, 32], as these areas are biomechanically vulnerable to trauma forces [46]. CTE is generally characterized by an irregular, patchy distribution of argyrophilic tau-immunoreactive neocortical NFTs [3]. When CTE increases in severity they become more densely distributed and are increasingly found across all brain regions, preferentially in layer II and the upper third of layer III [33], accompanied by increasing macroanatomical anomalies [32]. Additionally, tau-immunoreactive fibrillar astrocytic tangles and dot-like or spindle-shaped neurites have also been observed in the white matter as well as the basal ganglia and brain stem [3]. CTE originally described in the brains of boxers presented NFTs lacking Aβ plaques [47, 48]. More recently, other athletes presented focal p-tau abnormalities near focal axonal injury, alongside microhemorrhages, astrocytosis, and perivascular microgliosis, indicating a potential link to axonal injury and damage to the blood brain barrier [4, 49]. In parallel, military blast-related TBI and has revealed multiple areas of p-tau and glial immunoreactivity near small blood vessels [50–52]. The tau-containing neurons and neuritic thread clusters in the muskoxen in the present study are not as neuropathologically advanced as NFTs in human neurodegenerative disorders, and direct comparison of p-tau quantification between the current study and human CTE studies is not possible because of sampling from different regions with various methods. Nevertheless, their presence alone, in addition to the characteristic distribution pattern, is the first evidence of any form of naturally occurring brain trauma in bovids.

Abnormally phosphorylated tau has previously been detected using anti-pSer202/Thr205 tau in other aged animals, including bovids. Braak et al. [53] recorded abnormally phosphorylated tau in the allocortical regions of aged sheep and goats, with pSer202/Thr205 tau-immunoreactive neurons that resembled NFTs of early-stage AD. Additionally, Härtig et al. [54] reported two aged female American bison (*Bison bison*) as severely affected with abnormally phosphorylated tau in the prefrontal cortex [further explored in 55]. This could present a parallel to the female muskoxen in the present study which were also the most severely affected. Although presence of abnormally phosphorylated tau can be a sign of ageing, the severity of tau pathology in these bison, muskoxen, and bighorn sheep might also be related to headbutting, as the non-headbutting species in the Härtig et al. [54] study, such as reindeer, showed less severe tau-phosphorylation despite similarly advanced age. Other mammalian models present varying tauopathies when subjected to experimentally induced TBI. Histopathological analysis of mouse brains 24 h after lateral impact injury revealed dystrophic axons with hyperphosphorylated neurofilament proteins in proximity to reactive microglia and astrocytes [56]. Another mouse study detected pSer202/Thr205 tau immunoreactivity in the neocortex and hippocampus of animals subjected to mild repetitive TBI after four and ten weeks [57]. A similar experiment in rats showed an increase in p-tau expression in layers II/III of the motor cortex [58]. Using ferrets as a gyrencephalic TBI model, Schwerin et al. [59] demonstrated increased pSer202/Thr205 tau-immunoreactivity in the hippocampus after blast injuries, and a strong pSer202 tau-immunoreactivity in the superficial neocortical layers. In pigs where TBI was induced by rotational acceleration, Aβ and pSer396/Ser404 immunoreactive tau accumulated in axonal bulbs throughout the brains in addition to tau accumulations resembling pretangles in neuronal cytoplasm in the frontal, parietal, and temporal cortices colocalized with Aβ [60]. However, experimentally induced TBI is not directly comparable to naturally occurring TBI, especially for smaller model animals and depending on the methodology, additional damage is created through craniotomy [10], which can impede cellular investigation due to inflammatory and morphological change independent of TBI [61].

In humans, a single TBI leads to a high chance of developing neurodegenerative diseases later in life [62], and chronic TBI only increases those chances. Although little empirical data are available, muskoxen and bighorn sheep are known to headbutt every year during the rut. These bovids have a relatively short lifespan (muskoxen ♂: 10–12 years, ♀: 15–23 years approximately [63]; bighorn sheep ♂:10–12 years, ♀: 12–16 years approximately [64]) with an active reproductive period of 5–10 years in males. Each year the rut lasts from July to mid-October for muskoxen [22] and is slightly shorter for bighorn sheep [20]. It is uncertain how frequently individual males butt heads; however, single fights have been observed to comprise of 4–20 clashes, lasting multiple hours. A low estimate of three fights per week during the rut, with five clashes each, leads to about 210 clashes per year at around 60 km/h, averaging 2100 clashes in a lifetime. In comparison, studies on professional football players recorded a median number of 250 impacts per season [65] at around 20 km/h, similarly averaging 2000 clashes in a lifetime for an average career of 8 years, furthering headbutting bovids as a model for sports-related TBI.

The minimal p-tau immunostaining across bighorn sheep specimens could be attributed to the young age of the animals and the captive status of most of the specimens, impeding natural headbutting behaviors. Tissue degradation may have been an issue, as only one bighorn brain was injected with formalin before being shipped on ice overnight, while the muskox brains were preserved in formalin within hours after death. The limited immunoreactivity to pSer202/Thr205 tau and pSer202 tau in the bighorn sheep specimens could also indicate an absence of Serine 202 and Threonine 205 tau epitopes, as antibodies have less affinity for these epitopes in bovids, highlighting how tau physiopathology differs between species [66], which is further supported by an overall medium degree of homology between human and sheep tau protein reported in the BLAST protein analysis. Nevertheless, a human specimen with the same tau pathology present in the male bighorn sheep would have likely been diagnosed with mild CTE.

Another factor that may have contributed to the individual and species differences reported here is tau isoform expression shifting between different neurodegenerative diseases [67] and stages of NFT development. Anti-pSer202 tau and pSer202/Thr205 tau primarily recognize early and late NFT maturity levels, whereas anti-pSer396/Ser404 tau recognize more mature NFTs [38]. A lower detection by anti-pSer396/Ser404 tau and anti-pSer202/Thr205 tau in combination with the highest tau detection by anti-pSer202 tau in muskoxen suggests that all three individuals were in the early stages of neurodegeneration. Additionally, lack of reaction could be related to species differences in the structures themselves and trauma progression, in addition to different physiological processes of TBI in the early stages of pathology [38]. Additional discrepancies may include different tau sequences, protein folding, phosphorylation regulators, splicing and isoform expression, or other post-translational modifications of tau yet to be investigated in these species [68–75]. Despite most NFT maturation studies being focused on ageing and AD, many pathologies are shared with CTE and can be used as comparisons. For example, tau shows cellular and molecular changes specific to CTE cases, notably isoform signatures and 4R to 3R tau ratio in relation to CTE severity [76, 77].

The results of the present study offer an avenue of further research on brain trauma in wild animals. Different artiodactyls, including cetaceans [78], display different headbutting and sparring behaviors [10, 79], which present potential for further exploration of this phenomenon. As the pathology in our specimens appears to be in its early stages, future research should focus on whether further neurodegeneration is related with cognitive decline, as is the case in humans, where neurofibrillary degeneration can last more than 20 years. However, measuring cognitive function in bovids remains problematic, as no standardized behavioral tests exist for any bovid to date. The muskoxen’s capacity to survive yearly, repetitive, brain trauma and their similar frequency in this behavior to high-risk TBI individuals like football players and war veterans, presents them and likely other bovids including domestic sheep as a model with enough similarities to human CTE to explore the natural development of TBI. This is especially true given the translational difficulties of rodent studies.

In conclusion, this study has highlighted that muskoxen, bighorn sheep, and possibly other bovids, exhibit tauopathies in relation to TBI caused by headbutting. Our results indicate that while both sexes headbutt and develop related neuropathology, males may be better protected by their thick skull and horns. The subsistence of these animals despite chronic tauopathies is a distinctive evolutionary adaptation that encourages further investigation into the pathophysiology of TBI and CTE in the bovid model as an avenue for TBI prevention and treatment in humans.

## Supplementary Information

Below is the link to the electronic supplementary material.

Supplementary file1 (PDF 1632 kb)

## Data Availability

The dataset generated and analyzed during the current study is available in the supplementary information files provided as an online resource and on GitHub https://github.com/NLAckermans/Ackermans2022BovidTBI.git.
