# Supplementary material for: Evidence of traumatic brain injury in headbutting bovids
Source: Acta Neuropathol. 2022 May 17;144(1):5–26. doi: 10.1007/s00401-022-02427-2 (PMC9217783; doi:10.1007/s00401-022-02427-2)
Supplement: Supplementary file 1 — Supplementary file1 (PDF 1632 kb) [file 401_2022_2427_MOESM1_ESM.pdf]

Supplementary information -
Evidence of traumatic brain injury in headbutting bovids

Nicole L. Ackermans[1,2,5*], Merina Varghese[1,2], Terrie M. Williams[6], Nicholas Grimaldi[1,2], Enna Selmanovic[1,2], Akbar Alipour[2,4], Priti Balchandani[2,4], Joy S. Reidenberg[5], Patrick R. Hof[1,2,3]

[1]Nash Family Department of Neuroscience, [2]Friedman Brain Institute, [3]Ronald M. Loeb Center for Alzheimer's Disease, [4]BioMedical Engineering and Imaging Institute, and [5]Center for Anatomy and Functional Morphology, Icahn School of Medicine at Mount Sinai, New York, NY, USA
[6]Department of Ecology and Evolutionary Biology, University of California, Santa Cruz, CA, USA
* Corresponding author

Table S1: Exhaustive counts of different pSer202 tau-immunoreactive structures in the neocortical layers of the muskox brain.

Individual	Block	Region	Neuropil	NTC	Neuron
Male	PFC	I	679	130	1
		II	347	67	5
		III	585	92	4
		IV-VI	879	48	11
		WM	131	7	1
	parietal	I	299	1	0
		II	167	5	0
		III	293	15	0
		IV-VI	284	10	2
		WM	57	0	0
Middle-aged female	PFC	I	5557	680	2
		II	2515	319	21
		III	6205	743	25
		IV-VI	7089	838	38
		WM	4071	377	0
	parietal	I	417	5	0
		II	175	4	1
		III	321	9	0
		IV-VI	317	5	0
		WM	88	1	0
Old female	PFC	I	1658	44	0
		II	528	50	0
		III	2047	112	2
		IV-VI	776	36	3
		WM	314	20	0
	parietal	I	1316	49	0
		II	1494	78	3
		III	1420	68	1
		IV-VI	573	42	0
		WM	112	2	1

WM = white matter, PFC = prefrontal cortex

Table S2: Coefficient of error for estimated counts of pSer202 tau-immunoreactive structures structures in neocortical layers of the muskox brain.

Individual	Block	Region	Neuropil	NTC	Neuron
Male	PFC	I	0.04	0.09	1.00
		II	0.05	0.12	0.45
		III	0.04	0.10	0.50
		IV-VI	0.03	0.15	0.30
		WM	0.09	0.38	1.00
	parietal	I	0.07	1.00	NA
		II	0.08	0.45	NA
		III	0.07	0.26	NA
		IV-VI	0.07	0.32	0.71
		WM	0.14	NA	NA
Middle-aged female	PFC	I	0.03	0.04	0.71
		II	0.03	0.06	0.22
		III	0.02	0.04	0.20
		IV-VI	0.02	0.04	0.16
		WM	0.03	0.06	NA
	parietal	I	0.05	0.45	NA
		II	0.08	0.50	1.00
		III	0.06	0.33	NA
		IV-VI	0.06	0.45	NA
		WM	0.11	1.00	NA
Old female	PFC	I	0.03	0.15	NA
		II	0.05	0.14	NA
		III	0.03	0.09	0.71
		IV-VI	0.04	0.17	0.58
		WM	0.06	0.22	NA
	parietal	I	0.04	0.14	NA
		II	0.03	0.12	0.58
		III	0.03	0.12	1.00
		IV-VI	0.05	0.16	NA
		WM	0.10	0.71	1.00

NTC= neuritic thread cluster, WM= white matter, NA= no neurons counted,
PFC = prefrontal cortex

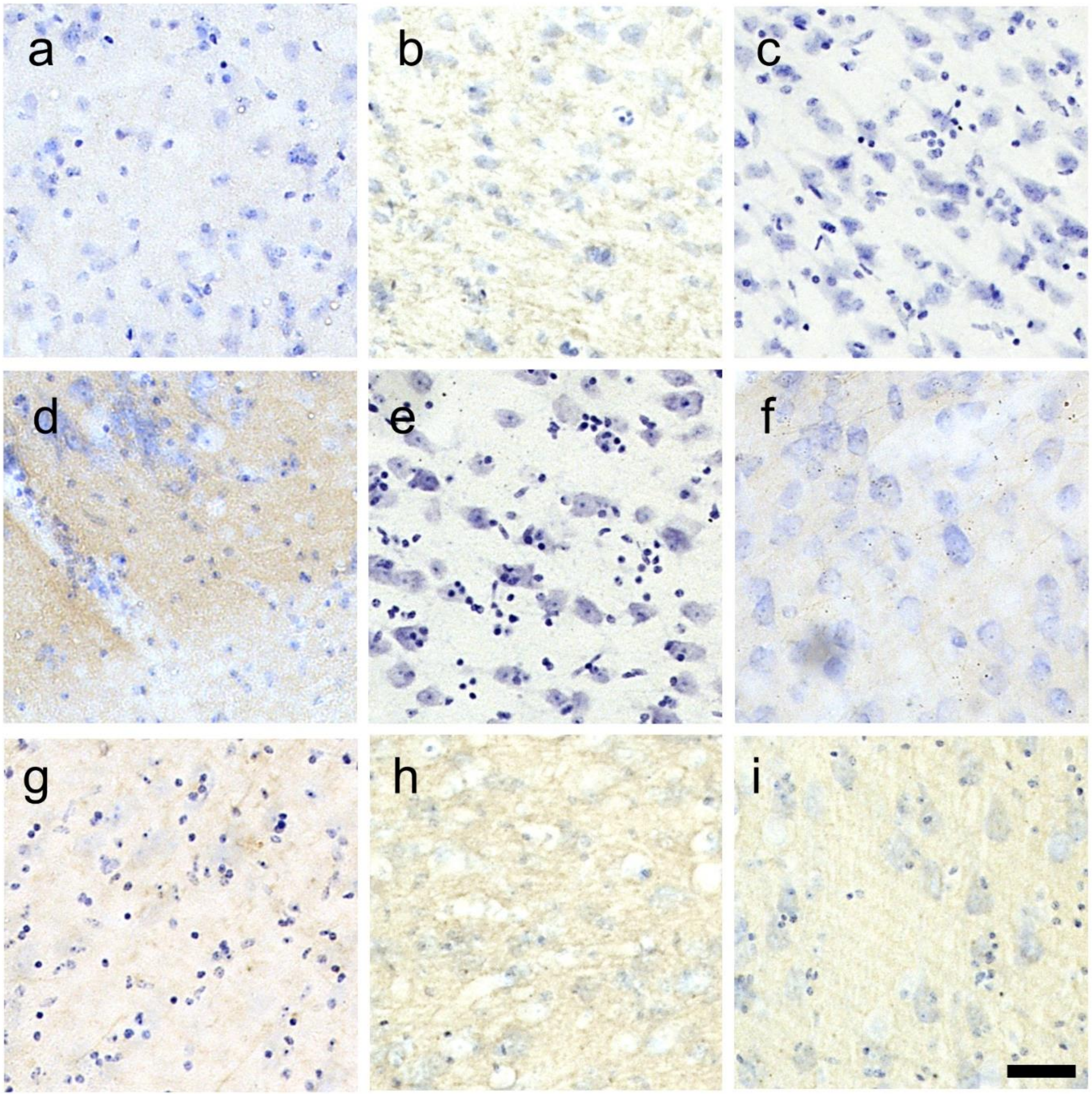

Figure S1. Photomicrographs of controls for abnormally phosphorylated tau immunostained for different antibodies in brains of human (a-c), old male muskox (d-f), and a male bighorn sheep (g-i). Samples were taken from the prefrontal cortex and subjected to immunohistochemistry in the absence of anti-pSer396/Ser404 tau (a, d, g), anti-pSer202/Thr205 tau (b, e, h), and pSer202 tau (c, f, i). The same control was used for both the Alzheimer's diseaseand CTE human specimens. Immunolabeled images can be found in Fig. 2. Scale bar = 100 µm.

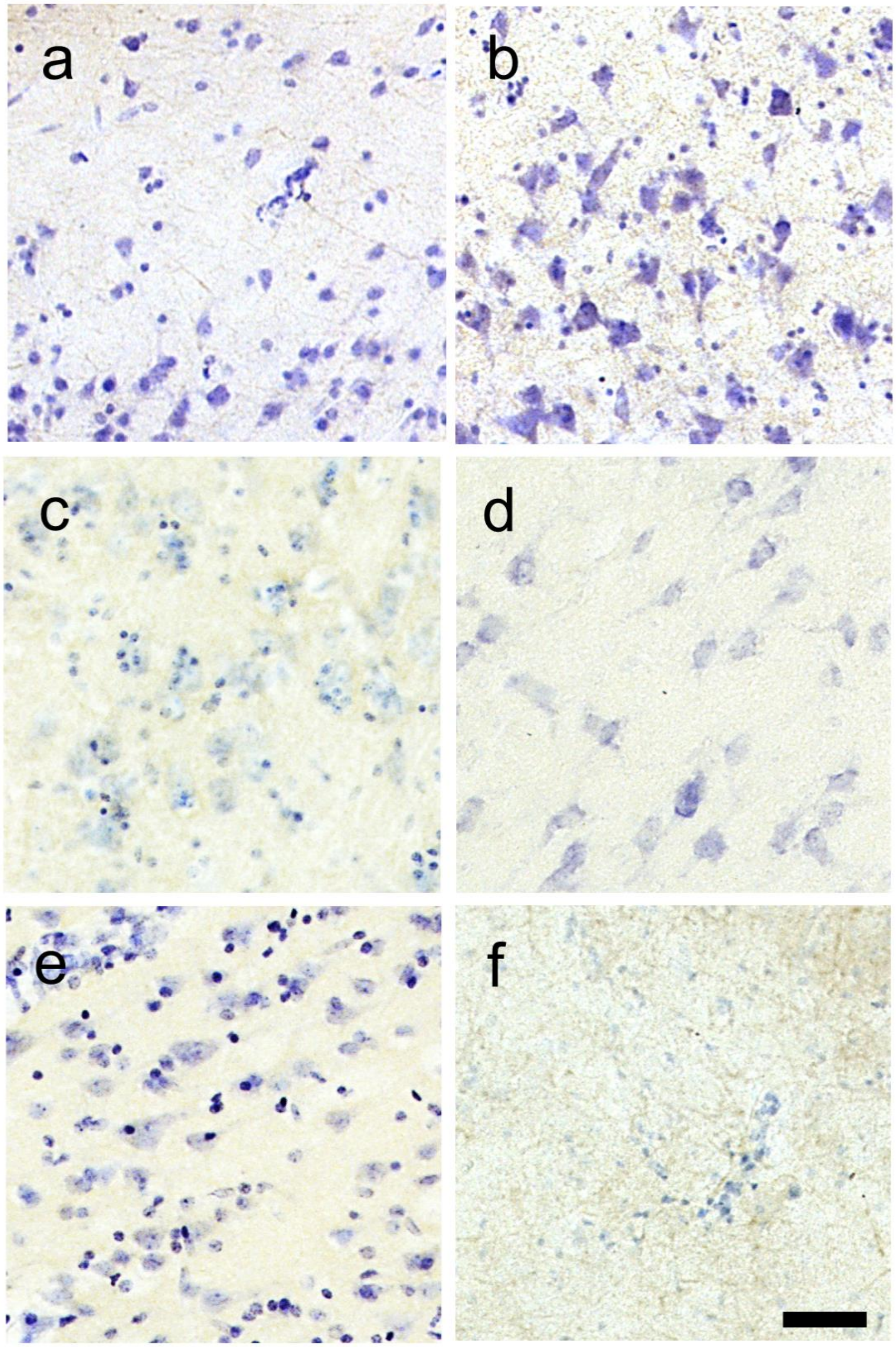

Figure S2. Photomicrographs of controls for anti-Iba1 (a-e) and anti-GFAP (b-f) immunohistochemical stain in brains of human with late stage CTE (a, b), an old male muskox (c, d), and a male bighorn sheep (e, f). Samples were taken from the prefrontal cortex and subjected to immunohistochemistry in the absence of Iba1 or GFAP. The same control was used for both the Alzheimer's disease and CTE human specimens. Immunolabled images can be found in Fig. 7. Scale bar = 100 µm.

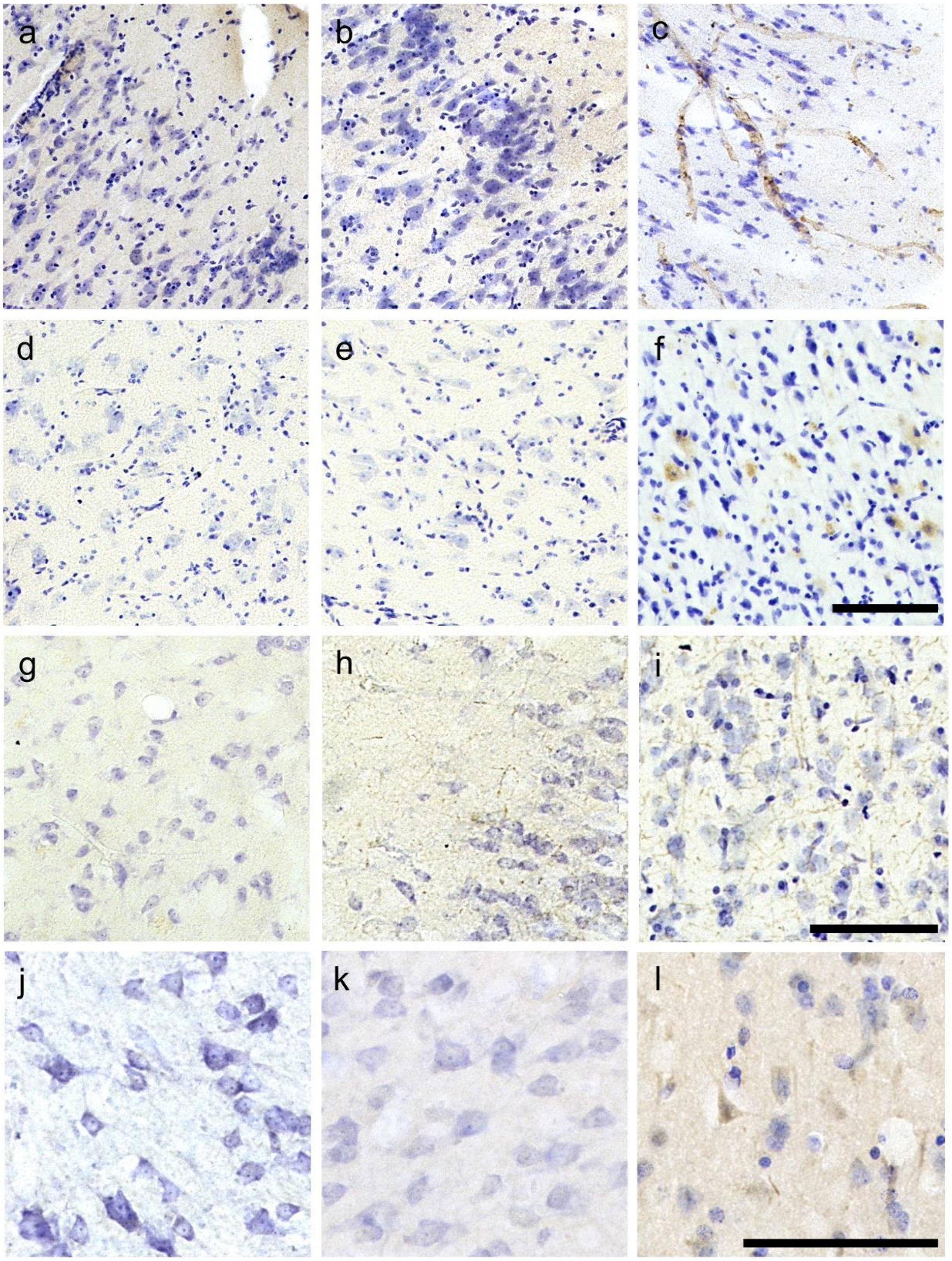

Figure S3. Immunohistochemical staining on muskox as compared to human brain tissue.
(a, d, g, j) Negative controls, (b, e, h, k) and light or no immunostaining in muskox prefrontal cortex, (c, f, I, l) immunostaining in the human CTE sample. (a-c) Anti-collagen IV antibody to highlight blood vessel morphology. Note immunoreactive blood vessels in (c) human tissue versus lack of labeling in (b) muskox. (d-f) Antibody Aβ for presence of Aβ. Note presences of plaques in (f) human tissue as opposed to (e) muskox tissue. (g-i) Antibody for degenerating myelin basic protein (dMBP). Note light immunostaining in both (h) muskox and (i) human tissue. (j-l) anti-pSer409/410 showed immunostaining in human tissue but not in muskox tissue. Scale bar = 100 μm.

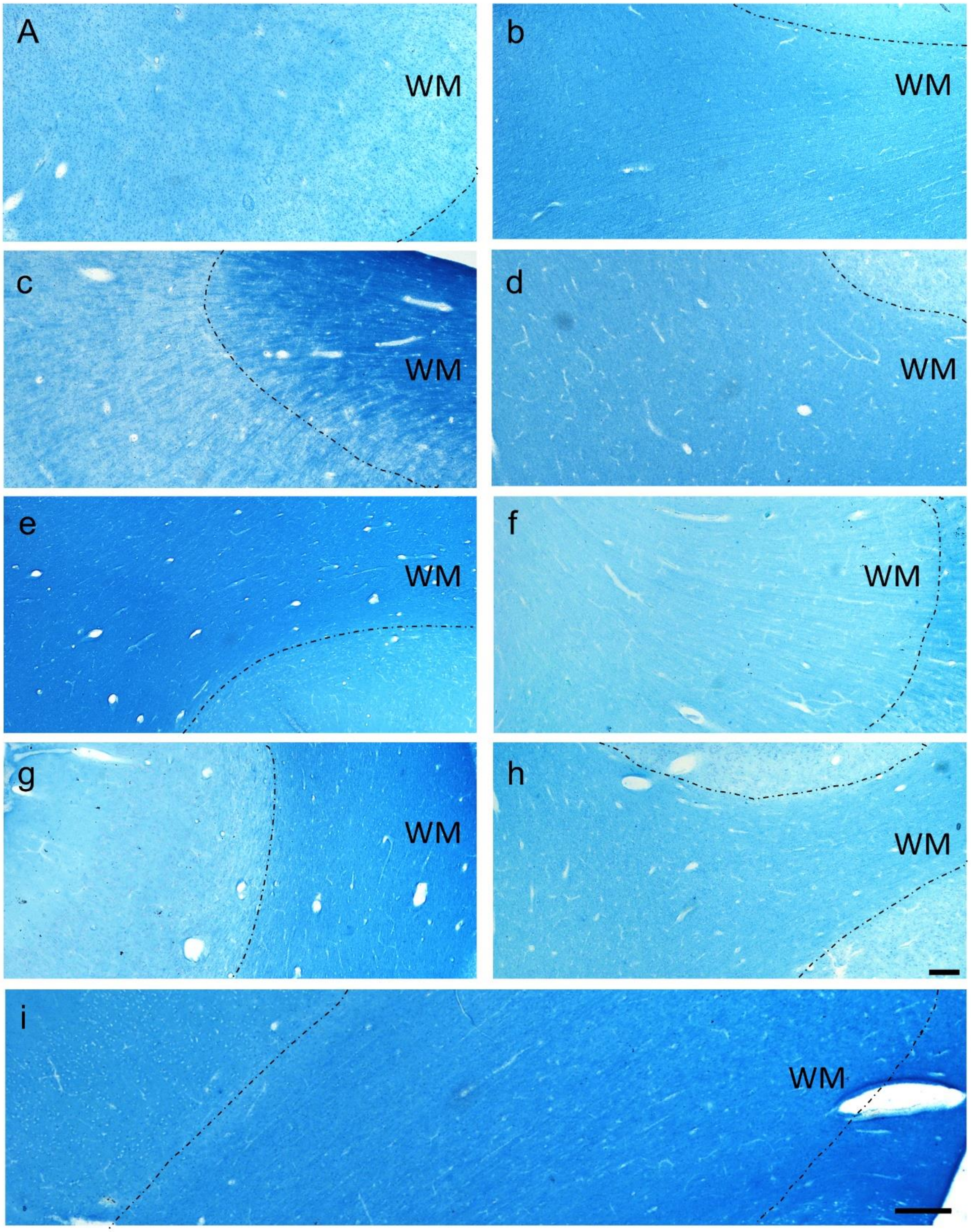

Fig S4. Photomicrographs of Luxol Fast Blue staining in human (a, b), muskox (c-h), and bighorn sheep (i) brain tissue to characterize myelination. (a) Human prefrontal cortex with advanced Alzheimer's disease with cloudy appearance of staining within the white matter. (b) Human superior frontal region with CTE, showing non-uniform staining in white matter. (c) Prefrontal cortex and (d) parietal regions of the old male muskox. (e) Prefrontal cortex and (f) parietal region of the middle-aged female muskox. (g) Prefrontal cortex and (h) parietal region of the old female muskox. (i) Prefrontal cortex of the male bighorn sheep. Dashed lines indicates white matter border, WM = white matter. Scale bar = 200 µm

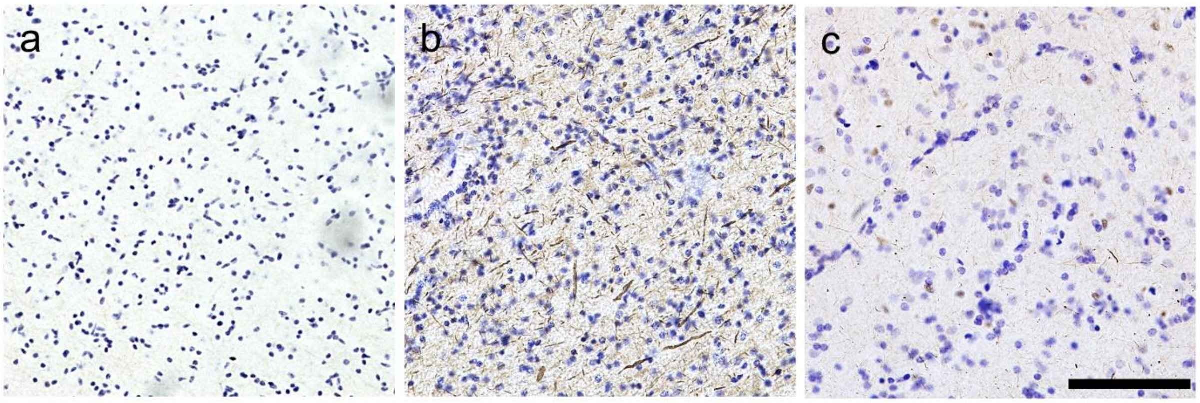

Figure S5. Photomicrographs of pNFP immunoreactivity using pNFP in muskox (a, b) and human (c). (a) Primary antibody control. (b) pNFP immunoreactivity in the old male muskox without any axonal anomalies. (c) pNFP immunoreactivity in a human sample without any axonal anomalies. Scale = 100 μm
